# On the Good Effects of Opium in Curing the Bite of the Snake, (*Coluber Berus, Linn.*)

**Published:** 1805-12

**Authors:** 


### On the good Effects of Opium in curing the Bite of the Snake, (*Coluber Berus, Linn.*) *By* Doctor Gassner.

A Country boy, eight years old, walking bare-footed in a forest, felt suddenly a pain at the middle of his left foot; soon after another near the condylus internus, which not regarding, he continued his walk, thinking it to be the effect of a nettle or thistle; but soon after he felt a third more violent pain at the tibia. Looking to the cause of it, he was frightened to find a snake winding herself round his leg, wounding these parts by different bites. Upon calling for assistance, he was carried home. During this time a swelling arose from the foot upwards to the integuments of the head of that side, so that he was unable to move any joint. He was often sick, and every fit of vomiting was accompanied with fainting.

Dr. G. found the patient cold, and the whole left side, from the face downwards, of various colours, blue, red, black, yellow, &c. hard like a stone, and swelled. The pulse trembling, and of such quickness, that it was impossible to count the pulsations; his head deranged, constantly sick; the lower jaw-bone drawn close to the upper one. Upon putting, with great difficulty, a little water between his lips, he suddenly became convulsed, and the tongue being thrown out, had a dark blue colour.

Dr. G. dilated the wounds, and ordered them to be washed with vinegar, and afterwards a blister to be put on. The swelled parts were rubbed with warm cloths, and afterwards with the tincture of opium. Round the neck and the lower jaw-bone a cataplasm of the leaves of henbane with the addition of opium was placed; and inwardly, wardly, twenty drops of laudanum Sydenhami prescribed. During twelve hours, 145 drops of laudanum were taken with very great trouble, when the vomiting diminished and the patient became a little more quiet. A slumber overtook him, during which the swelling abated, the warmth returned, and a perspiration was brought on, soon changing to a cold sweat. The laudanum was discontinued.—The swelling became more soft, the pulse rose, decreasing in quickness; and the next morning the swelling was almost entirely gone, and the patient in perfect possession of his senses. The blister had drawn a great quantity of water from the wounds; gradually the swelling entirely diminished, leaving only the colouring of the skin. By the use of camphorated spirit of wine, the spots were absorbed within eight days, and the patient perfectly recovered.

